# Translational and Emerging Clinical Applications of Medical Ultrasound

**DOI:** 10.1155/2018/6908393

**Published:** 2018-07-31

**Authors:** Yongjin Zhou, Weibao Qiu, Zhihong Huang

**Affiliations:** ^1^Shenzhen University, Guangdong, China; ^2^Shenzhen Institutes of Advanced Technology, Chinese Academy of Sciences, Shenzhen, China; ^3^University of Dundee, Dundee, UK

Wide and active usage of medical ultrasound can date back to the 1960s, yet it is not low-tech or obsolete. After decades of cooperative efforts from clinicians, researchers, and engineers, medical ultrasound is experiencing rapid developments in fields including optical-acoustic imaging, ultrasound elastography, advanced materials/technologies in ultrasonic transducers, ultrasound neuromodulation, ultrasound-guided interventions, and ultrafast ultrasound imaging. In this special issue on medical ultrasound, we have invited 13 papers that address translational and emerging clinical applications of medical ultrasound.

Specifically speaking, five papers in this special issue are more of novel engineering techniques in medical ultrasound. The only transducer paper titled “A High Frequency Geometric Focusing Transducer Based on 1-3 Piezocomposite for Intravascular Ultrasound Imaging” introduces a high frequency geometric focusing piezocomposite transducer, which can output high axial and lateral resolution and subsequently improve the imaging quality. Then two papers titled “A Modified 2D Multiresolution Hybrid Algorithm for Ultrasound Strain Imaging” and “A Normalized Shear Deformation Indicator for Ultrasound Strain Elastography in Breast Tissues: An* In Vivo* Feasibility Study” are both focused on ultrasound elastography. The first introduced a novel 2D multiresolution hybrid method for displacement estimation, to deal with the high compression scenario, while the second one proposed a normalized shear deformation indicator, which is proposed to boost breast lesion differentiation via a subsample speckle tracking algorithm. Another two papers titled “Machine Learning in Ultrasound Computer-Aided Diagnostic Systems: A Survey” and “Differentiation of the Follicular Neoplasm on the Gray-Scale US by Image Selection Subsampling along with the Marginal Outline Using Convolutional Neural Network” both apply machine learning/artificial intelligence in medical ultrasound. The first one is a review paper while the second is more specifically dealing with thyroid cancer.

Eight more papers are included in this special issue, covering various new clinical applications of medical ultrasound. Yet, four of them are more converged to musculoskeletal studies, while the others are more diversified for applications. Specifically speaking, the first paper titled “Correlation between Pathological Characteristics and Young's Modulus Value of Spastic Gastrocnemius in a Spinal Cord Injury Rat Model” is about the pathological characteristics and muscle stiffness of in rats with spinal cord injury using shear wave sonoelastography technology. The second paper titled “Automatic Myotendinous Junction Tracking in Ultrasound Images with Phase-Based Segmentation” aims at automatic localizing and tracking of the myotendinous junction, which is crucial to quantify the interactive length changes of muscle and tendon for understanding the mechanics and pathological conditions of the muscle-tendon unit during motion muscle contraction. The phase congruency was employed to perceive and enhance ridge-like features in the detection of tendinous tissues with Radon transform. The third paper titled “Gear Shifting of Quadriceps during Isometric Knee Extension Disclosed Using Ultrasonography” is also about image processing applications on muscle sonography, where it is found that quadriceps contract with a gear-shifting pattern under the protocol of isometric knee extension. The fourth and the last paper on muscle that is titled “Musculoskeletal Ultrasonography Assessment of Functional Magnetic Stimulation on the Effect of Glenohumeral Subluxation in Acute Poststroke Hemiplegic Patients” is more clinical oriented and presented the application of musculoskeletal ultrasonography on evaluation of the efficacy of functional magnetic stimulation (FMS) in the treatment of glenohumeral subluxation (GHS) in acute hemiplegic patients.

The last four papers are on prenatal diagnostics, cancellous bone, prostate cancer, and needle biopsy, respectively. The first one titled “Ultrasound in Prenatal Diagnostics and Its Impact on the Epidemiology of Spina Bifida in a National Cohort from Denmark with a Comparison to Sweden” is believed to cover the impact of prenatal ultrasound screening on the incidence of a major malformation on a national level. The second paper titled “Variability in Ultrasound Backscatter Induced by Trabecular Microstructure Deterioration in Cancellous Bone” investigates the relationship between ultrasonic backscatter and cancellous bone microstructure deterioration and indicated that the ultrasonic backscatter could be affected by cancellous bone microstructure deterioration direction. The third paper titled “The Use of Ultrasound Imaging in the External Beam Radiotherapy Workflow of Prostate Cancer Patients” provides an updated review on the status of prostate cancer ultrasound-guided external beam radiotherapy treatments. The last paper titled “Contrast-Enhanced Ultrasound Improves the Pathological Outcomes of US-Guided Core Needle Biopsy That Targets the Viable Area of Anterior Mediastinal Masses” presents results on ultrasound-guided core needle biopsy to harvest sufficient tissue with more cellularity that could be used for underlying ancillary molecular studies and improve the pathologic yield.

*Yongjin Zhou* *Weibao Qiu* *Zhihong Huang*

